# The effects of vegetarian diets on bone health: A literature review

**DOI:** 10.3389/fendo.2022.899375

**Published:** 2022-08-05

**Authors:** Alberto Falchetti, Guido Cavati, Roberto Valenti, Christian Mingiano, Roberta Cosso, Luigi Gennari, Iacopo Chiodini, Daniela Merlotti

**Affiliations:** ^1^ Experimental Research Laboratory on Bone Metabolism, Istituto di Ricovero e Cura a Carattere Scientifico (IRCCS), Istituto Auxologico Italiano, Milan, Italy; ^2^ Department of Medicine Surgery and Neuroscience, University of Siena, Siena, Italy; ^3^ Deparment of Surgery, Perioperative Medicine Unit, Azienda Ospedaliera Universitaria Senese, Siena, Italy; ^4^ Unit of Functional and Osteoarticular Rehabilitation, San Giuseppe Hospital, Istituto di Ricovero e Cura a Caratttere Scientifico (IRCCS), Istituto Auxologico Italiano, Piancavallo, Italy; ^5^ Department of Endocrine and Metabolic Diseases, Istituto di Ricovero e Cura a Caratttere Scientifico (IRCCS), Istituto Auxologico Italiano, Milan, Italy; ^6^ Department of Medical Biotechnology and Translational Medicine, University of Milan, Milan, Italy; ^7^ Department of Medical Sciences, Azienda Ospedaliera Universitaria Senese, Siena, Italy

**Keywords:** vegetarian diets, bone, fracture, bone density, nutrients, adults, elderly, review

## Abstract

In these recent years many people are adopting a vegetarian type diet due to the numerous positive health effects of this regimen such as the reduction of the incidence of many chronic disorders like diabetes, hypertension, obesity and cancer. However this diet is quite restrictive and so it could be possible to have a deficiency in some specific nutrients, increasing the risk of osteoporosis and fractures. Although there are conflicting results on the effects of the vegetarian diet on bone health and fracture incidence, it is always recommendable in vegetarian people to have an adequate intake of calcium and vitamin D, through an increased intake of supplements, natural and fortified foods, an adequate intake of protein, fruit, vegetables, as well as vitamin B12. The aim of this literature review is to revise the actual knowledge of the effect of some nutrients and vegetarian diets on bone health.

## Introduction

In these recent years many people are adopting a vegetarian type diet due to the numerous positive health effects of this regimen such as the reduction of the incidence of many chronic disorders like diabetes, hypertension, obesity and cancer (1–3). However this diet is quite restrictive and so it could be possible to have a deficiency in some specific nutrients such as calcium and vitamin D, thus leading to bone loss, osteoporosis and an increased risk of fracture (4). Vegetarians subjects exclude from their diet fish, meat and all their derivatives (5). Generally the classification of the vegetarian diet is based on the type of foods included or excluded. We talk about the lacto-ovo-vegetarian diet when dairy products and eggs are included, while a lacto-vegetarian diet includes only dairy products and finally the vegan diet which excludes all animal derivatives. However, we can find numerous heterogeneities among these diets also related to the personal choice of each individual. Vegetarian people usually have lower BMI, lower blood pressure and reduced serum levels of total and low density lipoprotein cholesterol (6). In this respect, vegetarian foods are healthy and seem to be able to reduce the incidence of obesity, hypertension, diabetes, ischemic heart disease, metabolic syndrome, CVD, and some types of cancers, due to the reduction of BMI values (3, 7–9). Moreover vegetarian diet confers a high fiber intake, which is associated with a decreased incidence of pancreatic cancer, and with a reduction of all cause of mortality, especially CVD mortality (10). Generally most vegetarians have a healthy lifestyle, but various studies indicate that vegetarian diets can also have a negative impact on bone health, partly related to a low BMI, but also to reduced intakes of vitamin B12, calcium and vitamin D (11–14).

It is well known that dietary habits may have implications also on muscle function (2). The different nutrient composition of vegetarian diet compared to an omnivorous diet, may alter physiological responses to physical exercise and influence physical performance. In particular, nutrient composition might alter the responses to physical exercise because the different macro- and micronutrient intake may alter cardiac output, mitochondrial function, substrate availability and oxygen carrying capacity (15). These effects, especially when they occur in elderly people, may impact on the age-related loss of muscle mass and strength that may result in sarcopenia. Sarcopenia is a muscle disorder characterized by low muscle strength and mass which increases the risk for frailty, falls, hospitalization, impaired recovery, and mortality (16). The possible effect of diet on frailty is still controversial but recent studies seem to indicate that adherence to diets characterized by high consumption of plant-derived foods and lower consumption of animal-derived foods could be able to reduce the risk of frailty in community−dwelling older adults (17). Nutrition play an important role for maintaining bone health through the life and to reach an adequate peak of bone mass during growth which may impact on bone strength along with other lifestyle factors and physical activity, reducing bone loss or fracture risk (18).

## Nutrients variability in vegetarian diets and their role on bone health

Bone is an active and dynamic tissue that needs sufficient nutrients for the processes of remodeling and mineralization (19).

**Figure 1:**
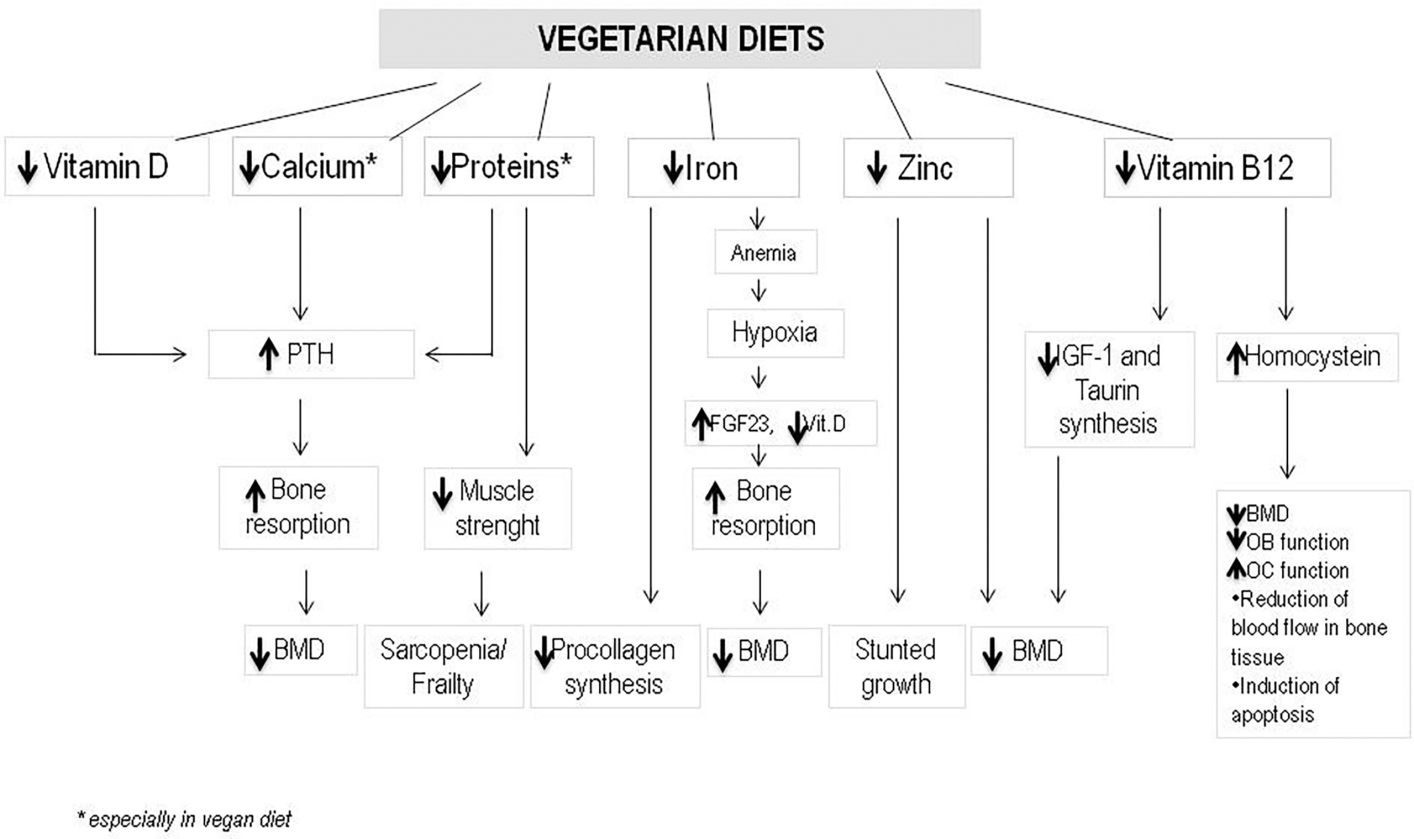
The different effects of nutrients variability in vegetarian and vegan diet on bone health. (PTH, Parathyroid hormone; BMD, bone mineral density; FGF23, fibroblast growth factor 23; IGF-1, insulin growth factor-1; OB, osteoblast; OC, osteoclast).

Dietary intake of some nutrients such as protein, Vitamin D, calcium, alcohol, or caffeine influences the regulation of bone remodeling (20). Vegetarian and vegan regimen diets have a reduced intake of calcium and proteins. Both these nutrients are essential for the maintenance but also for the development of bone mass and density. Therefore osteoporosis may affects both vegetarians and vegans more often than omnivores, which diet includes both vegetal and animal products. Additionally, bone health in vegetarians may be negatively influenced by other nutritional factors. Vegetarians often have lower consumption of zinc, phosphorus, vitamin B12, copper, which all have an effect on bone homeostasis (21). On the other hand, high quality vegetarian diet may include intakes of nutrients which protect bones, such as potassium (which lead to much lower acid load), Vitamin K, magnesium, some antioxidants such as Vitamins C and E and carotenoids, some anti inflammatory phyto-nutrients found in vegetables, fruits, legumes, nuts, tea, and herbs (22). The increased intake of fruits and vegetables leads to an increased amount of magnesium and potassium with positive effects on calcium and bone metabolism (23). Magnesium increases bone strength and influences calcium transport in the intestine (23). Vitamin K has been also associated with a protective affect on fracture risk (24). Nutrients and food in vegetarian diets and nutrients deficiency compared to omnivore are shown in **Tables 1**, **2**. The different effects of nutrients variability in vegetarian and vegan diet on bone health are showed in **Figure 1**.

**Table 1:** Nutrients and food in vegetarian diet.

Nutrients	Food
Protein	eggs, soy milk, soybean, soy products, tofu
Calcium	milk, cheese and yogurt, cabbage, mustard greens, broccoli, okra, legumes
Magnesium	tomatoes, spinach, legumes, beet, potatoes, raisins
Potassium	bananas, tomatoes, raisins, potatoes, spinach, papaya, oranges
Zinc	whole grains, beans, nuts
Vitamin C	broccoli, papaya, grapefruits, pineapple, oranges, strawberries
Vitamin K	collard greens, spinach, mustard greens
Vitamin B12	shiitake mushroom, yogurt, eggs, milk, cheese, nori

**Table 2:** Nutrient deficiency of different vegetarian diets (excluding fortified foods) compared to omnivores.

	Lacto-ovo-vegetarian	Lacto-vegetarian	Vegan
Calcium	No difference	No difference	Severe deficiency
Protein	No difference	No difference *	No difference *
Vitamin D	Mild deficiency	Mild deficiency	Severe deficiency
Iron	No difference **	No difference **	No difference **
Zinc	No difference	No difference	No difference
Vitamin B12	Mild deficiency	Mild deficiency	Severe deficiency

*different balance animal vs vegetal protein intake.

**despite a similar iron intake, vegetarian diet is associated with increased prevalence of anemia.

### Calcium and proteins

Calcium and proteins are generally consumed in form of dairy products and meat. Consequently, lacto-vegetarians do not have risk of calcium deficiency (21), while vegans consume substantially less calcium than other vegetarians and omnivores (1). Generally vegetarians should respect the same dietary indications and the same dietary intake references as omnivores to maintain bone homeostasis. As already mentioned, dairy products represent an important source of calcium from diet. However also various plant-based foods contain a good amount of absorbable calcium. These include broccoli, bok choy, tofu, kale and calcium-fortified foods such as fruit juices, energy bars and vegetable milks (25). The amount of oxalic acid and phytic acid, which could be found in some plant foods, is able to influence the levels of bioavailable calcium. Examples of foods rich in oxalic acid are rhubarb, spinach and chard; in these cases the absorption of calcium is highly reduced, equal to 5%, while for vegetables with reduced oxalic acid, such as broccoli and bok choy, the calcium absorption is 50% (26). Many studies have indicated that an increased dietary protein intake may increase the excretion of urinary calcium (27). Consequently, vegetarians, whose diet includes a reduced protein intake, should have reduced urinary losses of calcium and therefore need less calcium. Recently, several studies have indicated that the relationship between calcium balance and protein intake is much more complex and generally a diet rich in proteins is associated with positive effects on bone health, with an improvement of calcium absorption, especially in case of diets with a reduced calcium content (28, 29). Furthermore, proteins help to maintain bone structure and homeostasis by improving muscle strength and suppressing parathyroid hormone (PTH) (30). Vegetarians can found proteins in corn, soy, rice and wheat, which contain amounts of sulfates similar to what present in milk, meat and eggs (31). Moreover, the vegetarian diet involves a high intake of fruits and vegetables which are a good sources of potassium, calcium, magnesium, vitamin K and vitamin C; these nutrients are rich in antioxidants which could reduce the oxidative stress and bone resorption (32). The nutrients intake of vegetarians can vary according to food choices. Generally, the protein intake of non vegetarians is about 1% to 18% of the energy intake, whereas the protein intake in lacto-ovo-vegetarians and vegans is approximately 12-14% and 10-12%, respectively. Moreover, the type of diet, of course, influences the sources and the type of protein. A recent study on a population of Seventh Day Adventists showed that animal proteins is about 6.3% in non-vegetarians, 2.4% in lacto-ovo-vegetarians and 0.6% in vegans (33). Two recent studies have also compared nutrient intake between vegetarians and non vegetarians. The first study, named the European Investigation into Cancer and Nutrition (EPIC) 4- Oxford, evaluated the dietary intake of 29,913 meat consumers, 16,095 lacto-ovo-vegetarians and 2,112 vegans in the UK (34). The average percentage of protein energy was 16% in male meat consumers, 13.1% in male vegetarians, 12.9% in male vegans, 17.3% in meat users, 13.8% in vegetarians females and 13.5% in female vegans. Moreover, vegans showed higher intakes of magnesium and vitamin C but lower intakes of vitamins B12, D, calcium and zinc. The second study instead compared the intake of nutrients in different groups of adults belonging to the seventh day Adventist sect in the United States and Canada; 33,634 meat consumers, 21,799 lacto-ovo-vegetarians and 5,694 vegans were considered (33). The average protein intake, calcium, phosphorus, vitamin B12, sodium and zinc did not differ between the two groups. Furthermore, lacto-ovo-vegetarians and vegans had a significant higher intake of fiber; however vegans had a significant reduced intake of vitamin D and magnesium when compared with non vegetarians (p <0.05). A recent systematic review confirms that average protein intake is lower in vegetarians (13.4%) and vegans (12.9%) compared to meat eaters (16.0%), independently from the intake of supplements (14). Also calcium intake tends to be reduced in vegans with respect to vegetarians and meat eaters (14). Importantly a reduced intake of animal proteins could represent a big issue in specific populations, like patients with cancer, in which a balanced combination of animal and plant derived proteins is essential for supporting bone and muscle health and avoiding malnutrition in active cancer and during chemotherapy albeit a plant derived diet could be recommended in cancer prevention (2).

### Vitamin D

Vitamin D is able to modulate bone homeostasis by stimulating intestinal calcium absorption, promoting bone mineralization and maintaining muscle mass and strength (35). Vitamin D sources for vegetarians are represented by breakfast cereals, fortified plant-based beverages, fortified orange juice and fortified margarines. However, modest levels of vitamin D can be found also in mushrooms after exposure to ultraviolet light (36). Generally, dairy products are often fortified with vitamin D and they represent a good food source of this nutrient for lacto-ovo-vegetarians and lacto-vegetarians, while vegetable milk fortified with vitamin D, provides a source of this nutrient for vegans. However, these types of fortified foods are not easily available in Europe and elsewhere. For example, in Finland, during winter period, the dietary intake of vitamin D in lacto-ovo-vegetarians and vegans seems to be not sufficient to maintain both 25OH vitamin D and PTH levels in the normal range with possible negative effects on bone mineral density (BMD) (37). Various studies have also analyzed vitamin D status in vegetarians. The EPIC-Oxford study found significantly reduced levels of 25OH-vitamin D in vegetarian subjects compared to those eating meat while vegans had the lowest levels. Serum levels of 25OH-vitamin D equal to 25 nmol/l were found in 8% of vegans and 3% of vegetarians (13). The identification of an adequate dietary source of vitamin D is therefore necessary in vegetarians and vegans to maintain bone health and homeostasis. Although both fortified foods and UV-exposed mushrooms are widely used by vegetarian as plant sources of vitamin D, the amount they provide is not sufficient to guarantee the currently recommended RDAs of 600 IU/day for subjects from 19 to 70 years of age and 800 IU/day for subjects over 70 years of age, thus indicating that vitamin D supplementation in vegetarians is necessary. For low daily doses, both vitamin D2 and Vitamin D3 seems to be equally effective in maintaining circulating levels of serum 25OH-vitamin D (38). However, when given as a single dose, vitamin D3 appears to be more effective than vitamin D2 for increasing vitamin D levels (39). A recent meta-analysis on the effects of vitamin D-fortified foods on serum 25OH-vitamin D levels, markers of bone turnover (BTM) and BMD showed a significant increase in serum 25OH-vitamin D and BMD and a decrease in PTH levels (40). Thus, a vegetarian diet with appropriate food and supplements may provide a sufficient vitamin D intake and maintain a normal BMD (22).

### Vitamin B12

Vitamin B12 is essential for DNA synthesis, red blood cell formation, the myelination and function of the central nervous system and homocysteine metabolism (41). Vitamin B12 deficiency is quite common especially among elderly subjects and vegans who do not take supplements due to a reduced dietary intake of foods of animal origin. Generally the vegan dietary intake of vitamin B12 is below the daily recommended intake (DRI), while in lacto-ovo-vegetarians it can be variable according the use of dairy products (41). Vegans must obtain their vitamin B12 either from supplements or regular use of vitamin B12-fortified foods, such as breakfast cereals, vegetarian meat analogs, plant-based beverages. The introduction of unfortified plant foods such as leafy vegetables, algae (spirulina), fermented soy foods, mushrooms, and seaweeds, is not able to guarantee the daily recommended intake (DRI) of vitamin B12 (42). Other non animal sources of this vitamin are represented by fortified products like soy products, cereals and yeast. A deficiency of vitamin B12 may develop slowly in adult individuals. An adequate intake of vitamin B12 is important to prevent a sub-clinical deficiency that may go undetected along time. Generally, vitamin B12 deficiency is indicated by elevated serum levels of methylmalonic acid (MMA), while the serum vitamin B12 level is not a reliable indicator of vitamin B12 status (1). Vegetarians have reduced vitamin B12 levels and increased homocysteine ​​levels compared to non vegetarians. Recently a European study showed that vitamin B12 deficiency was present in 11% of omnivores, in 77% of lacto-ovo-vegetarians and in 92% of vegans when compared with omnivores (43). Moreover, to confirm this vitamin deficiency 5% of omnivores, 68% of lacto-ovo-vegetarians and 67% of vegans showed elevated serum levels of methylmalonic acid. Importantly, the negative effect of vitamin B12 deficiency on bone homeostasis may be complex. As first vitamin B12 deficiency may impact directly on taurine synthesis and insulin-like growth factor 1(IGF-1) production. However, vitamin B12 may also act through different mechanisms: 1) the reduction of bone mineral density and content 2) the reduction of osteoblasts function along with an increase of osteoclasts activity, 3) the reduction of blood flow in bone, 4) apoptosis induction through molecular pathways mediated by reactive oxygen-species (44). The relation between vitamin B12 deficiency and bone has not been deeply investigated to date. However, a recent observational study found that serum concentrations increase the risk of bone loss in patients with reduced levels of folate and vitamin B12 (45). Moreover a recent review indicates that average vitamin B12 intake is higher in meat eaters compared to vegetarians and vegans independently from supplements and similar results has been observed for what concern Vitamin B status (14).

### Iron and zinc

Iron may act as a co-factor for different enzymes involved in immune function processes, such as myeloperoxidase, and play an important role in amino acid metabolism and thyroid hormone synthesis (46). Generally omnivores have better iron status, with elevated concentrations of heme iron which is generally better absorbed. However, vegetarians, especially those with a well balanced dietetic regimen, are not at risk of iron deficiency. A correct iron intake is provided by a diet rich in seeds, wholegrain, legumes, green leafy vegetables, dried fruits, nuts and iron fortified cereal products. Generally, vegetarian diets may contain the same amount of iron than omnivore diets (34). However, anemia related to iron deficiency is more frequent in vegetarians than in omnivores (46). Iron plays an important function in many enzymatic pathways, including those involved in the process of collagen synthesis. Moreover, iron is able to regulate bone metabolism through the modulation of vitamin D functions. Concerning this point, the cytochrome P450 super family, which are monooxygenases containing heme, plays an important role (47). The relationship between iron and bone health derives from clinical studies in patients with iron overload associated with bone loss. The hypothesis of a possible relationship between bone and iron metabolism was described in some studies in which patients with disorders of iron metabolism, such as sickle cell disease, thalassemia, and hereditary hemochromatosis, showed an increased incidence of fractures and osteoporosis (48). In healthy populations, however, the relationship between bone metabolism and iron status is more controversial. Some studies indicated a positive correlation between BMD and serum ferritin in elderly men but not in women (49). In contrast, other studies found a negative association in women older than 45 years of age between BMD and either ferritin saturation or transferrin and no association in male subjects (50, 51). Thus, several mechanisms by which bone metabolism may be affected by iron deficiency, have been supposed. As we already mentioned iron is fundamental in vitamin D metabolism because it is an essential cofactor in the processes of hydroxylation of lysil and prolyl residues of procollagen. Another mechanism that can be involved is hypoxia, which is very frequent in anemic subjects in which oxygen supply to tissues is generally markedly reduced. It has been described that hypoxia is able to induce bone resorption, because it may increase osteoclasts activity which subsequently induces an increase in osteoblasts activation and function (52). Thus, it has been hypothesized that chronic iron deficiency may induce increased bone resorption and increase the risk of osteoporosis (47). Finally recent findings suggest that iron is able to regulate fibroblast growth factor 23 (FGF-23), a bone-derived hormone which plays an important role in phosphate homeostasis (53).

Zinc may act as a coenzyme for several enzymes which are involved in different processes like immunity, growth, bone function, regulation of gene expression and cognitive function (54). Zinc deficiency may cause stunted growth, reduced appetite, alopecia, dermatitis, impaired immunity and endocrine dysfunction (54). Zinc deficiency may be present both in vegetarians and in non-vegetarians (55). Phytates contained in cereals and legumes are able to reduce zinc absorption, while sprouting fermenting, or soaking reduce the levels of phytate making zinc more bioavailable (56). Vegetarian dietary sources of zinc include wholegrain, seeds, nuts, legumes, dairy products, tempeh, and tofu (57). The use of supplements and fortified breakfast cereals and foods may be essential for vegans (6). Some studies in postmenopausal women indicated that Zinc could be a possible marker of bone resorption (58). In fact urinary loss of zinc correlates with decreased bone mass and increased bone resorption (58). In addition, other studies in postmenopausal women showed significant associations between reduced concentrations of zinc, magnesium, iron and copper with reduced BMD (59). *In vivo* studies suggested that zinc may have a positive effect on the process of fracture healing after trauma both in animal models and in patients with fractures (60). Therefore, the importance of zinc supplementation for the maintenance of bone health has emerged. A recent observational study in elderly patients with osteoporosis and zinc deficiency showed that zinc supplementation may increase BMD and prevent fracture occurrence (61).

## Effect of vegetarian and vegan diet on bone mineral density (BMD) and fracture risk

Two important indicators of bone health are BMD, as assessed by dual x-ray absorptiometry (DXA) and impaired bone quality, which are responsible for the bone fragility and the risk of fracture. Various studies have examined these factors in vegetarians. It is well known that BMD is a good predictor of osteoporotic fracture risk (62). Various studies of BMD in vegetarians have reported conflicting and inconsistent results; some found no significant difference in terms of BMD, others reported reduced BMD values in vegetarians versus non vegetarians (63). These discrepancies may be attributable to the scarce number of cases examined, the differences between the types of vegetarian subjects studied and the lack of data on some factors such as physical activity, BMI, and nutritional intake. To better clarify these contrasting aspects, Ho-Pham et al. conducted a Bayesian meta-analysis to evaluate the effects of vegetarian diet on BMD (63). Nine different BMD studies in vegetarian subjects were considered, more than half in women. BMD in both the lumbar spine and the femoral neck was reduced by 4% in vegetarians (including both lacto-ovo-vegetarians and vegans) with respect to omnivores. Furthermore, the BMD was reduced by 6% at the femoral neck in vegans compared to non vegetarians with similar results also in the lumbar spine; however these differences were considered not clinically relevant in terms of fracture risk. We already mentioned that protein intake may be very variable in vegetarian and vegan diet according the food choice. Although various data confirm the negative role of protein deficiency on bone metabolism, a meta-analysis has shown that only 1-2% of BMD can be attributable to protein intake which can have both positive and neutral effects on BMD itself (64). Moreover another recent meta-analysis showed no difference between animal protein and soy on bone mineral density (BMD) and some markers of bone turnover (65). However, few studies have specifically examined the role of proteins in bone homeostasis in vegetarians. Recently, a cohort study of 1,865 peri- and postmenopausal women followed longitudinally for 25 years, evaluated the effects of eating meat or a vegetarian diet on wrist fracture risk (66). Vegetarian female subjects with the lowest intakes of vegetable proteins (beans, soy, soy milk, nuts and meat analogs) presented the highest risk of a wrist fracture. Moreover, a 68% reduction in this risk (HR: 0.32; 95% CI: 0.13, 0.79) was observed in vegetarian women who ate plant proteins more than once daily compared to those who ate 3 times per week; similar results were also present in those who consumed large quantities of beans, cheeses and meat analogs. A larger study of more than 17,000 vegetarian men and women showed that those with high intakes of meat analogs have a similar reduction in hip fracture risk to those with low intakes (HR: 0.34; 95% CI: 0.12, 0.95) (67). Recently, a cross-sectional study investigated the associations of veganism with BMD measured with calcaneal quantitative ultrasound (QUS), and also investigated the differences in the concentrations of different nutritional factors and bone related biomarkers between omnivores and vegans. This study showed lower levels of the QUS parameters in vegans compared to omnivores, with reduced levels of zinc, lysine, vitamin A, B2, selenium, protein P, urinary iodine, n-3 fatty acids and calcium levels, providing evidence of impaired bone homeostasis in vegans compared to omnivores, suggesting a relationship between different nutrition-related biomarkers and bone health (68). The EPIC-Oxford study instead examined the risk of fracture in consumers of fish, meat, vegetarians (who also ate eggs and dairy products) and vegans (69). In this study, 34,000 subjects aged between 20 and 89 were examined, followed for an average of 5.2 years and were asked whether there had been any previous fractures or not. The fracture risk was higher in vegans, although this association was partly reduced when the finding was corrected for non dietary factors such as alcohol and smoking. When, however, only subjects with reduced calcium intake were considered, there was no longer any difference between the various groups in terms of fracture incidence, thus suggesting that a correct calcium intake is fundamental for bone health regardless of other dietary habits (68). Recently the prospective EPIC-Oxford cohort study evaluated the fracture risk between vegetarians, non vegetarians and vegans (70). When compared with meat eaters and after adjustment for body mass index (BMI), socio-economic factors and lifestyle confounders, the risks of hip fracture were higher in fish consumers (hazard ratio 1.26; 95% CI 1.02–1.54), in vegetarians (1.25; 1.04–1.50), and in vegans (2.31; 1.66–3.22). Moreover, vegan subjects also showed higher risks of total fractures (1.43; 1.20–1.70), leg fractures (2.05; 1.23–3.41), and other main fractures (1.59; 1.02–2.50) than meat eaters. These risk differences were partly related to lower BMI, and presumably lower intakes of proteins and calcium (70). Other studies on postmenopausal Vietnamese women did not find significant differences in the risk of fracture at the vertebral level, comparing vegans with non vegetarians, while a greater risk of fracture at the wrist level was highlighted in a series of women. Therefore, vegetarians generally show a BMD similar to non vegetarians; likewise fracture risk does not differ if the calcium intake is adequate and the diet provides a correct protein intake. Furthermore, vitamin B12 deficiency, often present in vegetarians and particularly in vegans, has also been associated with reduced BMD and increased risk of fracture. Vitamin B12 deficiency, both mild and moderate, causes an increase in circulating levels of homocysteine, which is able to stimulate osteoclasts, inhibit osteoblasts and alter collagen crosslinks (71).

## Conclusions

The effects of a vegetarian diet on bone homeostasis have many implications. Reports and results may vary in different points such as populations size, study design and conclusions. Some studies showed significantly lower BMD in vegetarian subjects, especially vegans, which may explain the increased fracture risk, while other studies did not find any difference in bone health, suggesting that calcium and vitamin D intake is adequate for maintaining healthy bones and preventing fractures (72–74). In conclusion, although there are conflicting data on the effects of the vegetarian diet on bone health and fracture risk, in vegetarians it is always reasonable to follow some nutritional and dietary recommendations such as an adequate intake of calcium and vitamin D (through the intake of natural, fortified foods and supplements), an adequate intake of proteins and an abundant intake of fruit, vegetables, and vitamin B12.

## Author contributions

Conceptualization: DM and AF. Methodology: LG and RV. Data curation: DM, RV, GC, RC, CM, LG, IC, and AF. Writing—original draft preparation: DM, AF, and LG. Writing—review and editing: LG, IC. Supervision, DM, RV, AF and LG. All authors have read and agreed to the published version of the manuscript. All authors contributed to the article and approved the submitted version.

## Conflict of interest

The authors declare that the research was conducted in the absence of any commercial or financial relationships that could be construed as a potential conflict of interest.

## Publisher’s note

All claims expressed in this article are solely those of the authors and do not necessarily represent those of their affiliated organizations, or those of the publisher, the editors and the reviewers. Any product that may be evaluated in this article, or claim that may be made by its manufacturer, is not guaranteed or endorsed by the publisher.
